# Advances in the application of traditional Chinese medicine during the COVID-19 recovery period: A review

**DOI:** 10.1097/MD.0000000000037683

**Published:** 2024-04-05

**Authors:** Weixin Zhang, Linlin Ma, Wei Xie, Xingxing Li, Juhua Zhang, Ji Sun

**Affiliations:** a Collaborative Innovation Center for Biomedicines, Shanghai University of Medicine and Health Sciences, Shanghai, China; b School of Medical Technology, Shanghai University of Medicine and Health Sciences, Shanghai, China; c School of Pharmacy, Shanghai University of Medicine and Health Sciences, Shanghai, China; d Graduate School, Shanghai University of Traditional Chinese Medicine, Shanghai, China; e College of Nursing and Allied Health Sciences, St. Paul University Manila, Manila, Philippines.

**Keywords:** pharmacology, post-COVID-19 condition, recovery period, rehabilitation, traditional Chinese medicine

## Abstract

Since the emergence of the Coronavirus Disease 2019 (COVID-19) outbreak, significant advancements has been made in research, from limited knowledge about the disease to the development of a vaccine. Although the severity of Severe Acute Respiratory Syndrome Coronavirus 2 (SARS-CoV-2) appears to be decreasing and the threat of COVID-19 is waning, there have been widespread concerns about persistent symptoms or sequelae experienced by some patients even after recovering from COVID-19. Traditional Chinese medicine (TCM) has shown favorable treatment outcomes during the onset of COVID-19, and extensive studies have been carried out to explore the efficacy of TCM interventions during the COVID-19 recovery period. The purpose of this review is to comprehensively analyze these studies and provide new insights for the prevention and treatment of the post-COVID-19 condition.

## 1. Introduction

In December 2019, an outbreak of Coronavirus disease 2019 (COVID-19) occurred in Wuhan, Hubei Province, China.^[1,2]^ By March 11, 2020, the World Health Organization (WHO) had declared it a pandemic.^[3]^ As of October 4, 2023, there have been over 770 million confirmed cases and 6.9 million deaths reported globally.^[4]^ During the COVID-19 pandemic, much attention was given to the number of severe acute respiratory syndrome coronavirus 2 (SARS-CoV-2) infections, hospitalizations, and fatelities.^[5]^ However, there was less focus on assessing the risk of developing symptoms following the acute stage of SARS-CoV-2 infection.^[5]^ Evidence has revealed that certain COVID-19 patients continue to experience persistent symptoms or sequelae after “recovery.”^[6–8]^ The WHO has designated this condition as a new disease called post-COVID-19 condition.^[9]^

The main clinical manifestations of post-COVID-19 condition include pulmonary, cardiovascular, neuropsychiatric, renal, and dermatologic related symptoms.^[10]^ A Meta-Analysis and Systematic Review^[11]^ has demonstrated that the prevalence of the post-COVID-19 condition is high (with an estimated global pooled prevalence of 0.43) and that COVID-19 appears to have long-lasting health effects, which can put a strain on the healthcare system.^[11]^ As the number of patients who have turned negative from COVID-19 grows, more individuals are suffering from the post-COVID-19 condition. After more than 3 years of experience in COVID-19 prevention and therapy by healthcare professionals worldwide, attention is gradually shifting to this poorly understood condition.

**Figure 1.**
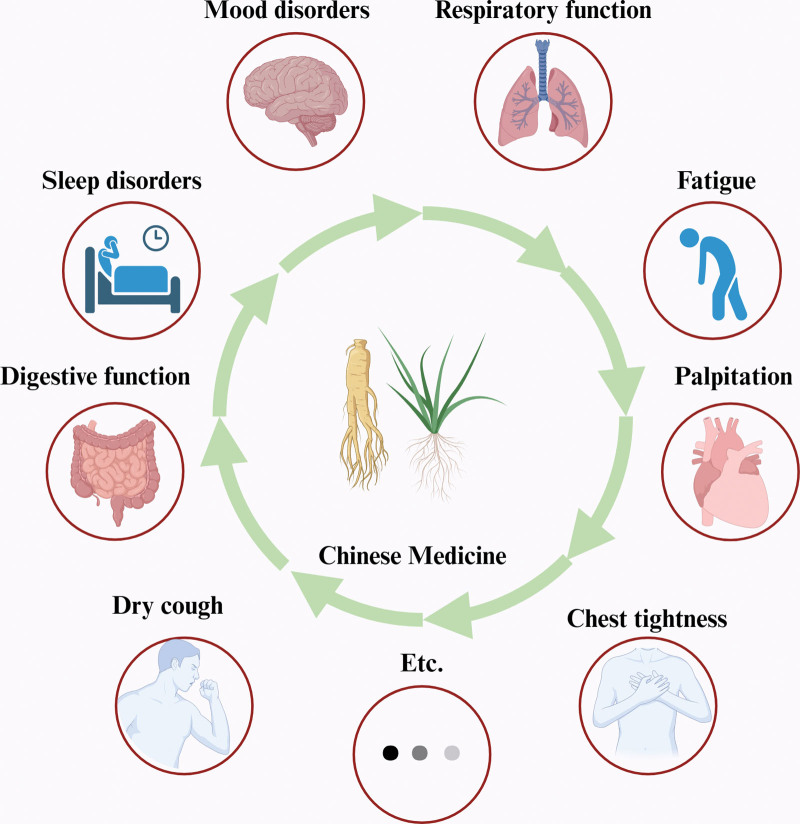
the application of TCM in the COVID-19 recovery period. Figure created with BioRender.com.

Traditional Chinese medicine (TCM), as an essential component of Asian culture, is a holistic system of medicine used for the diagnosis, prevention, and treatment of disease.^[12]^ TCM interventions have shown positive outcomes during the onset stages of COVID-19.^[13–15]^ The “three formulae and three medicines” including Huashi Baidu decoction, Qingfei Paidu decoction, Xuanfei Baidu decoction, Lianhua Qingwen capsules, Jinhua Qinggan granules, and Xuebijing injection has been shown to be effective in the COVID-19 onset phase.^[16]^ TCM rehabilitation therapy is currently under active investigation to address the post-COVID-19 condition.^[17–19]^ This article focuses on reviewing clinical studies with a relatively high level of evidence aimed at exploring the progress of research on the application of TCM during the COVID-19 recovery period (Fig. 1), with a focus on understanding the post-COVID-19 condition and the application of TCM in the COVID-19 recovery period. The goal is to provide novel insights into the prevention and treatment of the post-COVID-19 condition.

## 2. Post-COVID-19 condition

SARS-CoV-2 is a member of the same coronavirus family as Severe Acute Respiratory Syndrome Coronavirus (SARS-CoV) and Middle East Respiratory Syndrome Coronavirus.^[20]^ Survivors of Severe Acute Respiratory Syndrome (SARS) and Middle East Respiratory Syndrome often experience long-term effects such as lung function problems, psychological disturbance, and reduced exercise capacity.^[21]^ Similarly, some COVID-19 survivors have reported symptoms such as fatigue, pain, trouble breathing, loss of sense of taste or smell, arthralgia, anxiety, or depression.^[6–8,22–24]^

Several terms have been used to describe the long-term impact of COVID-19, including “Long COVID,”^[25]^ “Long COVID-19,”^[26]^ “post-acute COVID-19,”^[27]^ “post-COVID-19 syndrome,”^[28]^ etc. These terms have not been standardized, leading to confusion.^[29]^ To address this issue, the WHO has unified these definitions as the post-COVID-19 condition, which occurs in individuals with a history of probable or confirmed SARS-CoV-2 infection, typically 3 months after onset, and lasts for at least 2 months with symptoms that cannot be explained by an alternative diagnosis.^[9]^ Symptoms common include, but are not limited to, fatigue, shortness of breath, cognitive dysfunction, and impact daily functioning.^[9]^ Symptoms might be of new onset following initial recovery from an acute COVID-19 episode or persist from the initial illness, and they may fluctuate or relapse over time.^[9]^ Fatigue is a significant post-COVID-19 symptom.^[30–32]^ However, according to Rudroff et al,^[33]^ the understanding and definition of fatigue are not comprehensive, and there is wide variation in its definition. Therefore, post-COVID-19 fatigue is defined by them as “the decrease in physical and/or mental performance that results from changes in central, psychological, and/or peripheral factors due to the COVID-19 disease.”^[33]^

The COVID-19 Omicron variant exhibits a significantly higher percentage of asymptomatic infections and nonsevere disease than the Delta variant.^[34]^ Consequently, it has replaced Delta as the predominant epidemic variant of SARS-CoV-2.^[35,36]^ Additionally, a lower proportion of Omicron cases report the post-COVID-19 condition than Delta cases.^[37,38]^ However, this does not mean that the post-COVID-19 condition following Omicron infection should be ignored. According to a study^[39]^ of an artificial intelligence model trained on extensive experimental data and extensively validated by SARS-CoV-2 experimental results, The infectivity of Omicron may be over 10 times higher than that of the original SARS-CoV-2 virus and approximately 2.8 times higher than the Delta variant. Therefore, more individuals are likely to come into contact with Omicron. Given a large base of infections, the number of people affected by the post-COVID-19 condition is still huge, even if the incidence of such condition decreases.

## 3. Application of TCM in the recovery period of COVID-19

China’s National Health Commission and National Administration of Traditional Chinese Medicine have issued the Diagnosis and Treatment Protocol for COVID-19 Patients (Trial Version 10),^[40]^ which recommends the use of TCM prescriptions and acupuncture points during the COVID-19 recovery period. In addition, the same organization has issued the Recommendation on the Rehabilitation Guidance of Traditional Chinese Medicine for COVID-19 in the Recovery Period (Trial),^[41]^ which provides comprehensive recommendations for various TCM treatment methods, including Chinese medicines, acupuncture, moxibustion, tui-na, dietary guidance, and traditional Chinese exercises et al.

### 3.1. The recovery period of COVID-19 in the context of TCM

TCM views COVID-19 as a subtype of febrile diseases known as “plague,” and that SARS-CoV-2 can be understood as the “outcry” in the Wen Xie.^[42]^ TCM considers epidemic diseases arise from an external pathogenic attack, traditionally known as pestilent qi or evil qi, which is triggered by changes in the environment or weather.^[43]^ According to TCM principles, the primary pathogenic factor of COVID-19 is the accumulation of dampness in the lung, which infiltrates the body from the outside progressively affects various organs throughout the disease’s stages, from early onset to recovery.^[43]^

Yang et al^[44]^ considered that the WHO definition of post-COVID-19 condition primarily emphasizes its long-term implications, which may not be suitable for conducting early interventional studies in short-term conditions. TCM has always prioritized disease prevention, and over 2000 years ago, the ancient TCM text Huang Di Nei Jing proposed that “the upper doctor treats the untreated diseases, the middle doctor treats the desired diseases, and the lower doctor treats the existing diseases.” According to TCM theory, COVID-19 recovery period belongs to the rehabilitation stage of epidemic diseases. Rehabilitation stage of epidemic diseases represents a pattern stage when the disease enters the recovery period, the healthy qi has not been fully restored and the remaining evil remains unfinished.^[45]^ The basic pathogenesis of this stage is that the remaining evil is unfinished, or the “hidden toxin” is born inside, healthy qi deficiency and lingering pathogen.^[45]^ Intervention during the COVID-19 recovery period can be considered early prevention and treatment of the post-COVID-19 condition. In the disease’s developmental stage, when the anti-evil reaction occurs, treatment focuses on eliminating the pathogenic factors.^[45]^ With the proceeding treatment, the pathogenic qi gradually disappears into the recovery period, and the pathogenesis and disease location will change accordingly.^[45]^ This may be one of the explanations why some symptoms of the post-COVID-19 condition, for instance fatigue and joint pain, seem to be unrelated to respiratory or pulmonary infections.

TCM has historically focused on Bian Zheng Lun Zhi (Treatment based on pattern identification). Pattern identification is a process that involves a comprehensive analysis of clinical information obtained from the 4 major procedures of TCM diagnosis: observation, listening, questioning, and pulse analysis.^[46]^ It is used to guide the selection of appropriate TCM treatment using Fufang (acupuncture and or TCM herbal formulae).^[46]^ In 2020, the Recommendation on the Rehabilitation Guidance of Traditional Chinese Medicine for COVID-19 in the Recovery Period (Trial) provided recommendations for Chinese medicines to address both lung and spleen qi deficiency pattern and deficiency of both qi and yin pattern.^[41]^ In the latest treatment protocol released in January 2023, TCM recommendation for pattern of cold fluid retained in the lung were added, based on the old version the syndrome of lung and spleen qi deficiency pattern and deficiency of both qi and yin pattern.^[40,47]^ According to TCM theory, the lung and spleen are 2 internal organs that are related to each other as mother and son, which can influence each other.^[48,49]^ In the recovery phase of COVID-19, the lung qi is seriously depleted in the battle with the epidemic qi during the onset of the disease, resulting in clinical symptoms of lung qi deficiency.^[49]^ In addition, the son’s illness tiring mother, the lung qi deficiency cannot assist the spleen in transporting water and food essence.^[49]^ The son “stealing” the mother’s qi that causing damage to the spleen qi, thus, ultimately resulting in the pattern of lung and spleen qi deficiency.^[49]^ In the COVID-19 recovery phase, the healthy qi and yin liquid are severely depleted throughout the disease process, meanwhile, the toxic epidemic may still be present, the residual heat can still damage the lung and stomach qi and yin, resulting in a deficiency of both qi and yin pattern.^[49]^ The main syndrome of cold fluid retained in the lung is persistent cough. Content of cold fluid retained in the lung pattern was added to the Diagnosis and Treatment Protocol for COVID-19 patients (Trial Version 10) for patients who have more obvious cough symptoms during the recovery period.^[50]^

### 3.2. Chinese medicines application in the COVID-19 recovery period

In addition to the National Health Commission and National Administration of Traditional Chinese Medicine of China, the equivalent authorities of each Chinese province, municipality, and autonomous region have also developed their own COVID-19 diagnosis, treatment, and rehabilitation programs based on the principles of TCM San Yin Zhi Yi. This is applicable according to the TCM constitution, gender, age, and other differences of the patients, as well as the seasons and geographical environment, to develop appropriate treatment methods. Jiang et al^[51]^ analyzed the data from various COVID-19 diagnosis and treatment plans for the recovery period of COVID-19. The study showed that the core Chinese medicines of prescribed in the recovery period were Astragali Radix, Glycyrrhizae Radix et Rhizoma, Codonopsis Radix, Citri Reticulatae Pericarpium, Poria, Atractylodis Macrocephalae Rhizoma, Schisandrae Chinensis Fructus, Agastache rugosa, Glehniae Radix, Mori Folium, Gypsum Fibrosum, Amomi Fructus, Pinelliae Rhizoma, and Ophiopogonis Radix.^[51]^ During the COVID-19 recovery period, primarily choose the type of Chinese medicines that tonify qi, strengthen the spleen, and nourish yin, and the primary therapy approach was strengthening the body while removing pathogenic factors.^[51]^ Zhong et al^[18]^ treated 150 participants who were discharged after COVID-19 with TCM for 3 to 6 months according to the recommended prescriptions in the TCM clinical practice guidelines for COVID-19 patients, individual TCM syndromes, and clinical symptoms. The results show that the individualized TCM treatment facilitated the improvement of clinical symptoms and lung function and the achievement of a balanced body constitution in these patients recovering from COVID-19.^[18]^

Since the outbreak of COVID-19, many studies have been reported on the application of Chinese medicines in the recovery period thereof (Table 1). These studies explore the potential of Chinese patent drugs for the treatment of patients recovering from COVID-19, including new applications of existing medicines and the development of new ones. A project by An et al^[17]^ investigated the treatment effects of 6 already marketed Chinese patent medicines on 3 primary symptoms of COVID-19 recovery, namely cardiopulmonary dysfunction, sleep disturbance, and digestive dysfunction. The results demonstrated that Jinshuibao (JSB) tablets and Shengmaiyin oral liquid greatly improved patient cardiopulmonary function; Shumian capsules significantly improved sleep disorders; and Xiangshaliujun pills and Ludangshen oral liquid (LDS) significantly improved digestive function.^[17]^

**Table 1:** Study on the role of Chinese medicines in the recovery period of COVID-19.

Chinese medicine name	Chinese medicine type	Composition	Research method and sample size (intervention group, control group)	Intervention/Observation time (days)	Evaluation indicators	Main results	Ref.
Shumian capsules	Chinese patent drug	Semen Ziziphi Spinosae, Radix Bupleuri, Radix Paeoniae Alba, Flos Albiziae, Cortex Albiziae, Bombyx Batryticatus, Periostracum Cicadae, and Medulla Junci	Randomized, double-blind, placebo-controlled clinical study, n = 200 (G1 = 100, G2 = 100)	14/14	TCMSs	Improve sleep and mood disorders due to COVID-19	An X et al^[17]^ 2021
Ludangshen oral liquid	Chinese patent drug	Ludangshen	Randomized, double-blind, placebo-controlled multicenter trial, n = 200 (G1 = 100, G2 = 100)	14/14	Symptom VAS	Improve certain symptoms related to decreased digestive and respiratory functions	An X et al^[52]^ 2022
Bufei Huoxue capsules	Chinese patent drug	Astragali Radix, Paeoniae Radix Rubra, and Psoraleae Fructus	Multicenter, double-blind, and randomized controlled trial, n = 131 (G1 = 66, G2 = 65)	90/90	6MWD; chest CT; TCMSs; SGRQ; BDS; FAI	Reduces symptoms of fatigue, improves exercise tolerance	Chen Y et al^[53]^ 2022
Jinshuibao capsules	Chinese patent drug	Fermented Cordyceps	Pilot randomized, double-blind, placebo-controlled clinical trial, n = 200 (G1 = 100, G2 = 100)	14/14	Symptom VAS	Improvement in shortness of breath, sweating, chest tightness, dry cough, and palpitation	Yuehong Z et al^[54]^ 2023
Shengmaiyin oral liquid	Chinese patent drug	Ginseng, Ophiopogon japonicus, and Schisandra	Randomized, double blind, multicenter control trial, n = 200 (G1 = 100, G2 = 100)	14/14	Symptom VAS	Improve patient’s chest distress symptoms	Xuedong AN et al^[55]^ 2023
Shugan Jieyu capsule	Chinese patent drug	Hypericum perforatum L., Acanthopanacis Senticosi Radix et Rhizoma Sue Caulis	randomized, double-blind, placebo-controlled trial, n = 200 (G1 = 100, G2 = 100)	42/42	HAMD-17; HAMA; PHQ-15; ISI; TESS	Ameliorate sleep and emotional disorder	Xuedong AN et al^[56]^ 2022
Xiangsha Liujun pills	Chinese patent drug	Radix Aucklandiae, Fructus Amomi, Radix Codonopsis, Rhizoma Atractylodis Macrocephalae, Poria, Radix Glycyrrhizae Preparata, Pericarpium Citri Reticulatae, Rhizoma Pinelliae, Rhizoma Zingiberis Recens, Jujube (Chinese Date)	Randomized, double blind, placebo controlled clinical trial, n = 200 (G1 = 100, G2 = 100)	14/14	Symptom VAS	Ameliorate the symptoms related to the digestive function	Yuehong Z et al^[57]^ 2023
Qingjin Yiqi granules	Chinese patent drug	Ginseng Radix Et Rhizoma, Ophiopogonis Radix, Schisandrae Chinensis Fructus, Poria, Pinelliae Rhizoma, Scrophulariae Radix, Atractylodis Rhizoma, Citri Reticulatae Pericarpium, Glycyrrhizae Radix Et Rhizoma, Bupleuri Radix, Cimicifugae Rhizoma, Coicis Semen, Scutellariae Radix, Verbenae Herba, Phragmitis Rhizoma, and Lophatheri Herba	Randomized clinical trial, n = 388 (G1 = 194, G2 = 194)	14/14	mMRC; BS; 6MWD; Ss	Improves symptoms of breathlessness and fatigue	Pang W et al^[58]^ 2022
	Formulas	For the pathogen residue syndrome: Salvia miltiorrhiza 15 g, Prepared Fructus hordei germinates 30 g, Fructus hordei germinates 30 g, Codonopsis pilosula 15 g, Adenophora stricta 15 g, Peach kernel 6 g, Melon burdock 20 g, Magnolia officinalis 10 g, Radix Reed 30 g, and Herba patriniae 30 g For both qi and yin deficiency syndrome: Radix adenophorae 15 g, Ophiopogon japonicus 15 g, Astragalus membranaceus 15 g, Rhizoma Dioscoreae 15 g, and Massa Fermentata 10 g	Prospective cohort and nested case-control study, n = 96 (G1 = 64, G2 = 32)	28/84	CS; TCMS; chest CT	Improve lung inflammation	Li L et al^[59]^ 2021

6MWD = 6-min walk distance, BDS = Borg-Dyspnea Scale, BS = Borg scale, CS = clinical symptoms, CT = computed tomography, FAI = Fatigue Assessment Inventory score, HAMA = Hamilton Anxiety Scale scores, HAMD-17 = Hamilton Depression Scale total scores, ISI = Insomnia Severity Index scores, mMRC = modified Medical Research Council scale, PHQ-15 = Patient Health Questionnaire-15 scores, SGRQ = St. George’s Respiratory Questionnaire score, Ss = Symptoms score, TCMS = Traditional Chinese Medicine syndrome, TCMSs = Traditional Chinese Medicine syndrome score, TESS = Treatment Emergent Symptom Scale scores, VAS = visual analogue scale.

**Figure 2.**
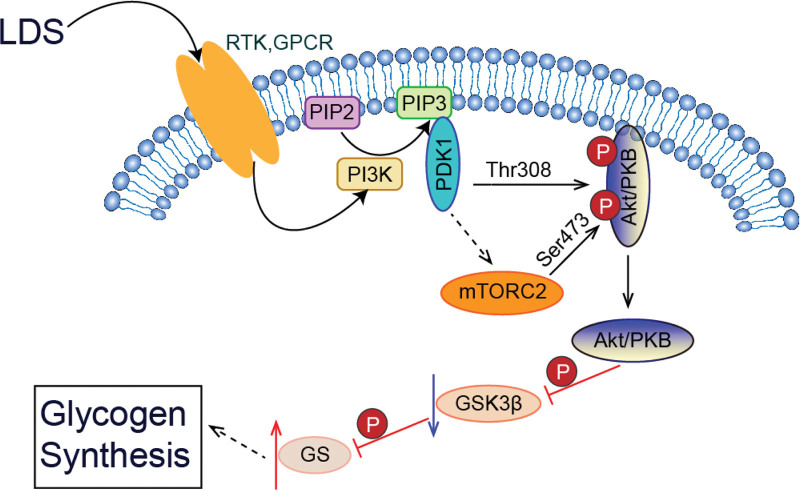
The effect of LDS on the PI3K/AKT/GSK3β signaling pathway.

LDS, which is made up of a better quality Codonopsis Radix known as Ludangshen, has the effect of replenishing the spleen and stomach qi, strengthening the spleen and lungs, and strengthening with tonics, originally used to treat spleen deficiency-type pediatric diarrhea, gynecological and obstetrical anemia, chronic gastritis, chronic nephritis, and both spleen and lung qi deficiency after radiotherapy.^[60,61]^ A randomized, double-blind, placebo-controlled multicenter trial explored the efficacy of LDS in patients recovering from COVID-19.^[52]^ The study showed that 2 weeks of treatment with LDS may have certain effects in improving symptoms of fatigue, anorexia, loose stools, and shortness of breath in patients recovering from COVID-19.^[52]^ LDS may be an option for the management of patients recovering from COVID-19 with digestive and respiratory symptoms.^[52]^ Codonopsis extract has a wide range of pharmacological actions, including neuroprotection, anti-aging and anti-oxidation, immune function regulation, and gastrointestinal function regulation, etc.^[62]^ Molecular mechanism research suggests that Ludangshen may activate the PI3K/AKT/GSK3β signaling pathway, promote the expression of p-PI3K, p-AKT, p-GSK3β, and GS, increase glycogen synthesis, thereby improving exercise endurance and alleviating exercise fatigue^[60]^ (Fig. 2).

**Figure 3.**
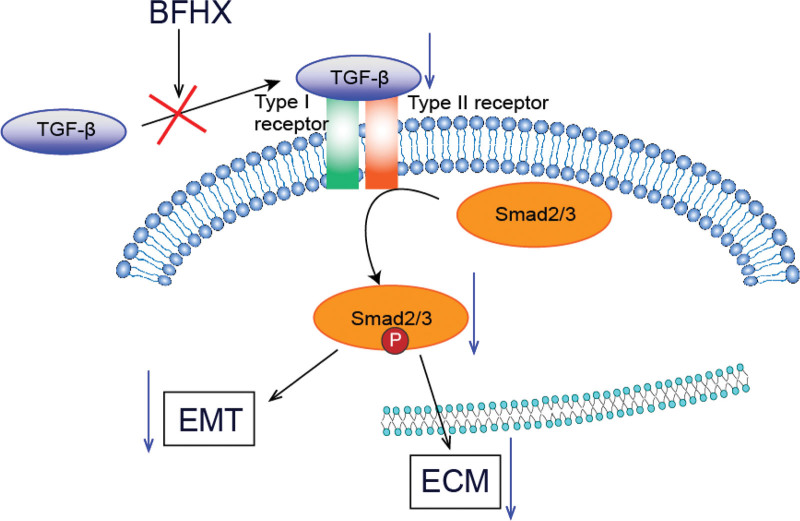
The effect of BFHX on the Smad2/3 signaling pathways.

Bufei Huoxue capsules (BFHX), composed of Astragali Radix, Paeoniae Radix Rubra, and Psoraleae Fructus, originally used to treat pulmonary heart disease, BFHX is used to invigorate the lung, strengthen the spleen, consolidate the kidney, replenish qi, and activate blood circulation.^[63,64]^ In a multicenter, double-blind, randomized controlled trial conducted by Chen et al,^[53]^ BFHX was found to reduce fatigue symptoms and improve exercise tolerance in patients recovering from COVID-19. They believed that taking BFHX will greatly improve symptoms and facilitate recovery in discharge patients with symptoms of fatigue, residual lung damage, and impaired exercise tolerance during COVID-19 recovery.^[53]^ The core compounds (quercetin, kaempferol, 7-O-methylisomucronulatol, baicalein and formononetin, etc.) contained in the BFHX can act on the targets of IL6, MAPK8, PTGS2, PTGS1, NCOA2, etc., regulating multiple signaling pathways, exercising their therapeutic effects on the recovery period of COVID-19 through the ways of antiviral, anti-bacterial, anti-inflammatory, and immune-modulation.^[65]^ In response to TGF-β1-induced epithelial-mesenchymal transition and extracellilar matrix, BFHX blocks phosphorylation of Smad2/3 signaling pathways by suppressing TGF-β1 activation^[66]^ (Fig. 3). According to the molecular docking results, the binding energies of the core compounds in the BFHX with SARS-CoV-2 3CL hydrolase and ACE2 were close to those of the currently used antiviral drugs lopinavir, ridecavir, and ritonavir^[65]^ It is hypothesized that the core compounds in the BFHX may prevent viral invasion of the host by binding to the ACE2 to prevent viral invasion of the host, and to control viral replication by binding to 3CL hydrolase.^[65]^

**Figure 4.**
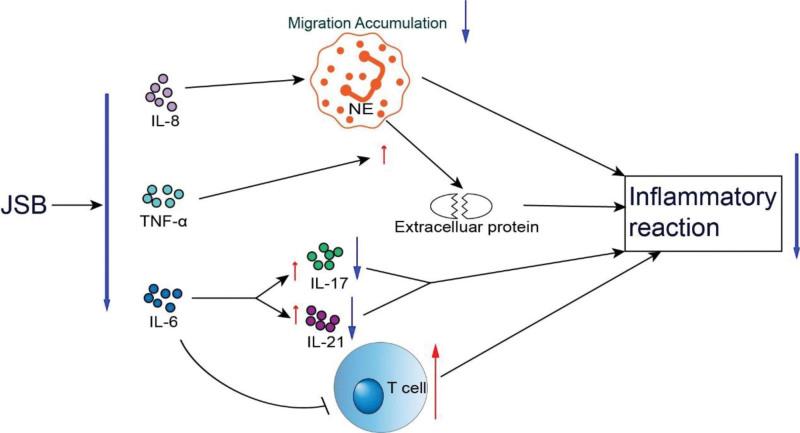
The effect of JSB on the inflammatory reaction.

JSB capsules are made of fermented Cordyceps sinensis powder,^[67]^ which has the effect of tonifying the lungs and kidneys, secreting essence and benefiting qi.^[68]^ It is a substitute for Cordyceps sinensis, a famous traditional Chinese medicine.^[69]^ A pilot randomized, double-blind, placebo-controlled clinical trial study showed that JSB capsules was effective in improving residual cardiopulmonary symptoms such as dry cough and palpitation in recovering COVID-19 patients.^[54]^ JSB reduces the levels of pro-inflammatory factors (IL-6, IL-8, and TNF-α), thereby alleviating and reducing the inflammatory response^[70]^ (Fig. 4). Cyclic administration of JSB Capsules can reduce the inflammatory response in the patient’s body, reduce oxidative stress, maintain the balance of bone metabolism, improve the endocrine level in the body, regulate the intestinal flora which will shorten the recovery time of the patients in the recovery period of COVID-19, as well as alleviate the functional abnormalities or functional deterioration of the respiratory, endocrine, and skeletal systems and other related sequelae.^[71]^

**Figure 5.**
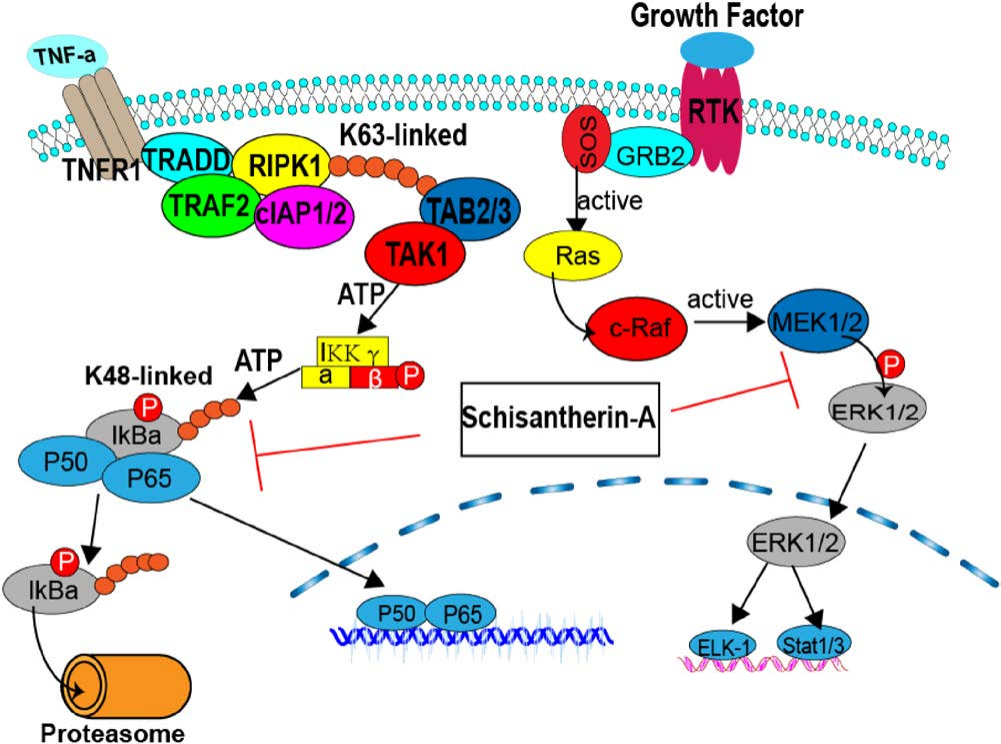
The effect of Schisantherin-A on the NF-κB and MAPK signaling pathways.

Shengmai Yin, a renowned herbal prescription documented in Qian Jin Yao Fang, consists of Radix ginseng, Radix ophiopogonis, and Fructus schisandrae.^[72,73]^ Shengmai Yin has demonstrated effectiveness against various ailments, including diabetes, myocardial infarction, heart failure, and viral myocarditis.^[73]^ Moreover, Shengmai Injection (Shengmai decoction), as a proprietary Chinese medicine, is recommended for critically ill COVID-19 patients in the Guidelines on the Diagnosis and Treatment of Novel Coronavirus-Infected Pneumonia. Several studies have indicated the efficacy of Shengmai in convalescent COVID-19 patients.^[55,74]^ Network pharmacology analysis has unveiled its molecular mechanism, which involves inhibiting inflammatory reactions, promoting antiviral activity, and regulating immunity. Key targets such as EGFR and MAPK1, as well as signaling pathway such as PI3K-Akt and MAPK signaling pathway, may be involved in its regulatory effects.^[74]^ Schisantherin-A is the key active compounds of SMI in the treatment of COVID-19.^[74]^ Schisantherin-A blocks activation of MAPK signaling pathways by suppressing the phosphor pation of ERK. In resting cells, NF-κB family members are sequestered in the cytoplasm, for they are bound to their inhibitors which belong to the IκB family. Upon cell activation, IκB proteins are phosphorylated and degraded by the proteasome. Subsequently, NF-κB could enter the nucleus and bind to the DNA, resulting in promoting transcription. Schisantherin-A restrained IκB-α degradation and NF-κB p65 activation. In conclusion, NF-κB and MAPK signaling pathways can be suppressed by schisantherin-A to regulate the release of pro-inflammatory cytokines, such as TNF-α, IL-6 and IL-1β^[75]^ (Fig. 5). Additionally, Shengmai Yin ranks among the top 3 formulas with the highest positive NESs (normalized enrichment scores), suggesting an immune-activating effect^[76]^ Similarly, Shugan Jieyu capsule^[56]^ and Xiangshaliujun pills^[57]^ were shown to be effective in trials for certain symptoms in patients recovering from COVID-19 (Table 1).

In addition to exploring new uses of existing drugs, new drugs have been developed for the sequelae of COVID-19. A randomized clinical trial by Pang et al,^[58]^ that included 388 patients with post-COVID-19 condition, was conducted to evaluate the effectiveness and safety of a new prescription called Qingjin Yiqi granules (QJYQ). The intervention group showed better improvement in the modified Medical Research Council scale and Borg scale data compared to the control, and no treatment-related adverse events were observed, this suggest that QJYQ is a safe Chinese medicine that can alleviate post-COVID-19 condition symptoms include breathlessness and fatigue.^[58]^ The main components of QJYQ are baicalin, naringin, malic acid, wogonoside, hesperidin and glycyrrhizic acid et al^[77]^ As a new Chinese patent medicine, the mechanism of action of QJYQ is being further investigated. Recent studies have shown that baicalin, schisandrin, ginsenoside Rb1, naringin, hesperidin, liquiritin, liquiritigenin, glycyrrhizic acid, and hastatoside pathological conditions rats in vivo processes are changed, which suggests that these substances may have pharmacological effects as active ingredients.^[78]^

In addition, in terms of Chinese Medicine Formula. Li et al^[59]^ conducted a prospective cohort study with a nested case-control design, where the intervention group received Chinese Medicine Formulas based on TCM pattern. The study demonstrated that TCM could effectively improve patient’s lung inflammation, leading to early recovery.^[59]^

## 4. Discussion

Traditional Chinese exercises, including Tai Chi, Liu Zi Jue, and Ba Duan Jin, are based on the principles of the unity of man and nature, the combination of hardness and softness, and combination of dynamic and static.^[79]^ These exercises can improve body functions, prevent diseases, strengthen the body, and relieve fatigue by regulating the body, breath, and mind, balancing yin and yang; and harmonizing the zang-fu organs and meridians and collaterals.^[79,80]^ Traditional Chinese exercises has been considered to improve the quality of life and relieve related symptoms in patients with chronic and pulmonary diseases.^[81,82]^ Among them Tai chi has been proven to be an appropriate substitute for pulmonary rehabilitation in chronic obstructive pulmonary disease.^[83]^ Since the outbreak of COVID-19, researchers have also been actively exploring the rehabilitative effects of traditional Chinese exercises on the COVID-19 recovery period, including randomized controlled trials on the effect of Tai Chi or Liu Zi Jue interventions on symptom or function improvement.^[84]^ Liu Zi Jue is a moderate-low intensity traditional Chinese exercise that combines breathing and simple body movements to strengthen bodily functions.^[85]^ A multicenter, prospective, self-controlled study by Tang et al^[86]^ demonstrated that Liu Zi Jue exercise produces superior functional capacity and quality of life in discharged COVID-19 patients, that it could be used as a substitute home exercise program.

According to a study by Han et al,^[87]^ which utilized a bioinformatics/network topology strategy, acupuncture against COVID-19 symptoms mainly through reducing inflammation, activating immunity, and modulating the nervous system. In the COVID-19 recovery period, a data mining study^[88]^ analyzed 77 acupoint prescriptions in the COVID-19 recovery period and identified Feishu (BL13), Zusanli (ST36), Qihai (RN6), Guanyuan (RN4), Pishu (BL20), Zhongwan (RN12), Neiguan (PC6), Tianshu (ST25), Sanyinjiao (SP6), Taiyuan (LU9), and Danzhong (RN17) as core acupoint prescription in the recovery period. These are primary tonifying points that can regulate the organs of pulmonary, spleen, kidneys, and heart of patients during the recovery period to reinforce healthy qi and eliminate any remaining pathogenic factors.^[88]^ A case report^[89]^ also showed that acupuncture at the Yingxiang point (LI20), which supposedly improve the sense of smell, may have a possible positive effect on olfactory dysfunction in the post-COVID-19 condition.

In Chinese, acupuncture (zhēn) and moxibustion (jiū) are collectively referred to as zhēnjiū, while moxibustion, as an acupuncture-related therapy,^[90]^ has been studied for its efficacy in the recovery phase of COVID-19. In a clinical study conducted by Tao et al,^[91]^ moxibustion was combined with TCM dialectical remedies to treat patients with both lung and spleen qi deficiency syndrome during COVID-19 recovery. The observation group, which received moxibustion in addition to TCM remedies, was compared to the control group, which only received TCM remedies.^[91]^ The results demonstrated that the observation group showed better TCM symptom score, St. George’s Respiratory Questionnaire score, total treatment efficiency, pulse oxygen saturation level, and 6 minute walking distance data than the control group.^[91]^ Moxibustion was found to be effective in improving the TCM clinical symptoms and promoting the recovery of patients with lung and spleen qi deficiency during the COVID-19 recovery period.^[91]^

A variety of TCM treatments are commonly utilized in combination with Western medicine to achieve improved outcomes. A clinical study conducted by Sun et al^[92]^ demonstrated that the intervention group treated with TCM comprehensive rehabilitation therapy combined with modern respiratory rehabilitation therapy significantly better than the control group treated with modern respiratory rehabilitation therapy and placebo in improvement of TCM symptom score. Another clinical study by Li^[93]^ also demonstrated that the combination of traditional Chinese and Western rehabilitation therapy, along with conventional therapy, was superior in mitigating COVID-19 related symptoms in patient compared to who received conventional therapy alone.

Regarding the mechanism of action of TCM, a retrospective analysis performed by An et al^[94]^ found that TCM treatment led to a decrease in white blood cell count, serum interleukin-6, procalcitonin, and serum γ-glutamyl transpeptidase, while prealbumin, albumin, red blood cell, hemoglobin, and platelet count were increased in patients recovering from COVID-19 compared to not TCM treatment. The mechanism of action of TCM may involve balancing the immune response, improving hematopoiesis and coagulation, enhancing liver and heart function, as well as increasing nutrient intake and lipid metabolism.^[94]^ Overall, TCM treatment seems to have unique advantages in treating COVID-19 recovery syndrome, a non-acute, multi-organ, and systemic disease that affects multiple organs.

Although numerous clinical studies have been conducted on TCM as a potential treatment for patients recovering from COVID-19, the academic community still demands further high-quality studies, as reflected in several relevant study protocol systematic reviews published,^[95–100]^ which highlights the insufficient number of existing studies. The modernization and globalization of TCM would benefit greatly from additional high-quality studies.

## 5. Conclusion

In the recovery period of COVID-19, TCM has demonstrated promising therapeutic effects, similar to its effectiveness in treating the acute onset of COVID-19, TCM interventions have shown alleviation of symptoms in patients recovering from COVID-19. TCM has its own unique understanding of the different symptoms associated with the post-COVID-19 condition. Utilizing this understanding to guide the choice of TCM treatment can have a beneficial intervention effect. Nevertheless, further studies are still necessary to strengthen the monitoring and observation of indicators, thereby providing more compelling clinical evidence.

## Acknowledgments

We thank Charlesworth Author Services (cwauthors.com) for its linguistic assistance during the preparation of this manuscript. Figure 1 were created with BioRender.com.

## Author contributions

**Conceptualization:** Ji Sun.

**Data curation:** Linlin Ma, Juhua Zhang.

**Funding acquisition:** Linlin Ma, Juhua Zhang, Ji Sun.

**Visualization:** Wei Xie, Xingxing Li.

**Writing – original draft:** Weixin Zhang.

**Writing – review & editing:** Linlin Ma, Juhua Zhang, Ji Sun.
